# Dermoscopy Differential Diagnosis Explorer (D3X) Ontology to Aggregate and Link Dermoscopic Patterns to Differential Diagnoses: Development and Usability Study

**DOI:** 10.2196/49613

**Published:** 2024-06-21

**Authors:** Rebecca Z Lin, Muhammad Tuan Amith, Cynthia X Wang, John Strickley, Cui Tao

**Affiliations:** 1 Division of Dermatology Washington University School of Medicine St. Louis, MO United States; 2 Department of Information Science University of North Texas Denton, TX United States; 3 Department of Biostatistics and Data Science The University of Texas Medical Branch Galveston, TX United States; 4 Department of Internal Medicine The University of Texas Medical Branch Galveston, TX United States; 5 Department of Dermatology Kaiser Permanente Redwood City Medical Center Redwood City, CA United States; 6 Division of Dermatology University of Louisville Louisville, KY United States; 7 Department of Artificial Intelligence and Informatics Mayo Clinic Jacksonville, FL United States

**Keywords:** medical informatics, biomedical ontology, ontology, ontologies, vocabulary, OWL, web ontology language, skin, semiotic, web app, web application, visual, visualization, dermoscopic, diagnosis, diagnoses, diagnostic, information storage, information retrieval, skin lesion, skin diseases, dermoscopy differential diagnosis explorer, dermatology, dermoscopy, differential diagnosis, information storage and retrieval

## Abstract

**Background:**

Dermoscopy is a growing field that uses microscopy to allow dermatologists and primary care physicians to identify skin lesions. For a given skin lesion, a wide variety of differential diagnoses exist, which may be challenging for inexperienced users to name and understand.

**Objective:**

In this study, we describe the creation of the dermoscopy differential diagnosis explorer (D3X), an ontology linking dermoscopic patterns to differential diagnoses.

**Methods:**

Existing ontologies that were incorporated into D3X include the elements of visuals ontology and dermoscopy elements of visuals ontology, which connect visual features to dermoscopic patterns. A list of differential diagnoses for each pattern was generated from the literature and in consultation with domain experts. Open-source images were incorporated from DermNet, Dermoscopedia, and open-access research papers.

**Results:**

D3X was encoded in the OWL 2 web ontology language and includes 3041 logical axioms, 1519 classes, 103 object properties, and 20 data properties. We compared D3X with publicly available ontologies in the dermatology domain using a semiotic theory–driven metric to measure the innate qualities of D3X with others. The results indicate that D3X is adequately comparable with other ontologies of the dermatology domain.

**Conclusions:**

The D3X ontology is a resource that can link and integrate dermoscopic differential diagnoses and supplementary information with existing ontology-based resources. Future directions include developing a web application based on D3X for dermoscopy education and clinical practice.

## Introduction

Dermoscopy is a noninvasive, in vivo microscopic technique used to examine skin lesions by detecting morphological features that may not be seen by the naked eye [1-4]. Studies have demonstrated that dermoscopy improves the diagnosis of both pigmented skin lesions [3,5-7] and nonpigmented skin lesions [8], including neoplasms [9] and infectious and inflammatory skin diseases [4,10]. Notably, the diagnostic accuracy of dermoscopy is dependent on the examiner’s experience, as dermoscopy by untrained or less experienced examiners was found to be no better than clinical inspection without dermoscopy [6]. Learning dermoscopy is not just relevant to dermatologists, but also for physicians in other medical specialties. Patients with new or changing skin lesions often first consult their primary care physician (PCP) rather than a dermatologist. Dermoscopy has shown to be an effective tool for the assessment and triage of pigmented skin lesions in primary care, with improved diagnostic accuracy and referral accuracy to dermatologists [11-13]. However, dermoscopy training for PCPs is currently highly variable, with many PCPs citing a lack of training as a key barrier to the use of dermoscopy [14-16]. Furthermore, short dermoscopy training programs [14] may be insufficient to establish long-term competency in dermoscopy, with poor continuing use of dermoscopy and the need for refresher sessions [17]. The need for dermoscopy training among plastic surgeons has recently been documented as well [18]. Thus, the development of machine-based tools for dermoscopy may enhance clinical practice for dermatology providers and other medical professionals.

The use of standard terminologies organized through taxonomies has a long history with the life sciences, starting with Carl Linnaeus’ taxonomy [19]: a classification system to name and group species according to their shared characteristics. Centuries later and with advances in computing infrastructure, these types of classification systems have continued to be of interest to the science community. An ontology is “a representational artifact comprising a taxonomy as proper part, whose representational units are intended to designate some combination of universals, defined classes, and certain relations between them” [20]. Essentially, an ontology is a graphical representation of linked concepts to formalize a schema (Tbox) for data (Abox). The formalization leverages semantic links (Rbox) between the concepts to give data more meaning and to aggregate related data of any heterogeneous format. This ensures the normalization of heterogeneous data. Furthermore, with semantics, ontologies could support machine reasoning to generate references via deductive reasoning. As related to the medical field, ontologies can extend the computability of standard controlled terminologies to provide descriptive and composite representations of medical information (such as features related to various diagnoses). Ontologies represent the data in a machine-readable format to give computing tools more context, making them highly valuable for artificial intelligence.

Within the dermatology domain, some existing ontologies aim to describe cutaneous disorders. For example, the dermatology lexicon (DERMLEX) was created with the American Academy of Dermatology with a nosology, anatomical distributions, classical signs, and therapeutic procedures; however, maintenance was discontinued in 2009 [21,22]. More recently, the human dermatological disease ontology (DERMO) was developed to classify cutaneous diseases by etiology, anatomical location or cell type, and phenotype consistent with current clinical practice [23,24]. Some other dermatology-specific ontologies exist, including the skin physiology ontology (SPO; last updated in 2008) [25], but notably, none of these ontologies connect cutaneous disorders to metaphoric terms like “strawberry pattern” which may be difficult for a machine to understand. Similarly, none of the aforementioned ontologies specifically address dermoscopy, which is a specialized technique that may have special considerations when used in diagnosis. For instance, the colors of certain lesions are best seen under polarized light [26]. As such, there is a need to develop an ontology that adequately addresses the field of dermoscopy, with the capability of processing both descriptive and metaphoric terminology.

In our previous work, we developed the elements of visuals ontology (EVO) to decompose the fundamental features of visualizations, such as shapes, colors, and textures. The dermoscopy elements of visuals ontology (DEVO) then applied the visual features described in EVO to dermoscopic terminology [27]. For instance, DEVO characterizes dermoscopic metaphoric terms such as “shiny white streaks” and “leaflike areas” by shapes, colors, and textures, along with other features involved. Discussion with domain experts revealed that while DEVO is capable of responding to queries to find visual features associated with metaphoric terms and vice versa, linking the dermoscopic terms to differential diagnoses would significantly enhance its clinical utility. A list of differential diagnoses indicates many possible diagnoses that share similar features to the patient’s symptoms and signs. These differential diagnoses can then be narrowed down to aid the clinician in identifying the final diagnosis. As dermatology is a technical field, the landscape of differential diagnoses is wide and difficult to parse [28]. In this study, we describe the extension of EVO and DEVO to create the dermoscopy differential diagnosis explorer (D3X), an ontology linking metaphoric terms to differential diagnoses. We further propose a use case integrating D3X into a web application in dermoscopy education and clinical practice.

## Methods

### Ethical Considerations

This article adheres to the Committee on Publication Ethics guidelines. This research did not involve human subjects.

### Integration of Existing Ontologies

#### Overview

A common practice in the development of ontologies [29] is to reuse existing ontologies’ components to ensure semantic interoperability. We used the following ontologies to build the D3X ontology.

#### About EVO

EVO is a foundational ontology model that describes the basic constituents of visualizations: shapes, colors, strokes (lines), size, perceived texture, etc. It also represents the dimensional extended 9-intersection model, a mathematical model for spatial relationships between elements [30]. Further, EVO imports and reuses controlled terminologies and standards from the W3C scalable vector graphics, Wikidata, phenotype and trait ontology, and the simple knowledge organization system to supplement our core representational model of visualizations. EVO is hosted on GitHub for public release and is coded in the OWL 2 web ontology language.

#### About DEVO

DEVO is an extension of EVO that reuses the foundational understanding of visualizations for the dermoscopy domain. DEVO incorporates some of the controlled terminologies—“metaphoric” and “descriptive”—that are used in practice by dermatologists, with a focus on the metaphoric terminologies. With DEVO, we developed a core model that encodes and describes the “visual language” of the dermoscopic terms’ definitions. Further, one important outcome of this work was a computable representational model of an agreed understanding of visual elements of dermoscopic patterns, which we used as a framework to generate differential diagnoses. Similarly, DEVO was coded in OWL 2 and is hosted on GitHub for public consumption.

#### Miscellaneous Ontologies and Vocabularies

We also aligned D3X with commonly used top-level ontologies. The information artifact ontology (IAO) [31] is part of the open biological and biomedical ontology (OBO) foundry. IAO represents a general abstraction of informational objects (like documents and components within those documents—eg, figures, images). Like many OBO foundry ontologies, IAO uses the basic formal ontology and relation ontology as part of its architecture model. We minimally reused some of the term entities and properties like IAO:image and “denoted by.” We also reused the software ontology (SWO) [32] for its licensing entity terms—SWO:license and “has license”—to describe the licensing information for any imaging resource of skin lesions. Lastly, we used Schema.org’s [33] schema::image to link image resources.

### Development of D3X

To generate a list of differential diagnoses, we started with the metaphoric terms defined in DEVO from the third consensus conference conducted by the International Society of Dermoscopy [34]. We then searched the literature [34-36] for corresponding differential diagnoses for each term and consulted 2 domain experts to independently edit the list of diagnoses for accuracy. These differential diagnoses were later encoded using Protégé [37] in our ontology. Following this, we reviewed open-source resources (DermNet, Dermoscopedia, and open-access research papers) for a collection of hosted images that matched individual differential diagnoses. We tracked the provenance information and associated data (caption, description, etc) in a spreadsheet as a central organized resource that mapped the images for each diagnosis to the concept diagnosis used in D3X. To streamline the data transfer process, we developed a management code to transfer data from the spreadsheet to the ontology. The source code is available on our GitHub repository, using the OWL API to facilitate efficient custom import. This approach allowed centralized data collection and also enabled an ad-hoc import and data creation pipeline.

### Semiotic Evaluation

Semiotic theory is the study of signs and symbols, and considering ontologies are symbolic representations of a specific domain, we used a metric suite grounded in that theoretical framework [38]. Semiotic theory is composed of 3 basic qualities: *syntactic, semantic*, and *pragmatic*. Essentially, in the context of ontologies, the metric suite components refer to aspects of the ontology artifact—*syntactic* concerning encoding adherence and standards; *semantic* concerning the effective use of human-friendly labels for entities and concepts; and *pragmatic* concerning function. Each of these qualities is quantified based on a computation of representative quantifiable features of an ontology file (eg, the number of classes, the average number of word senses for labels, etc). This is described in detail in previously published works [38]. This suite helps to measure some of the intrinsic qualities of our ontology concerning other ontologies in the same domain. We used publicly available ontologies from the skin and dermatology domain—DERMLEX, DERMO, and SPO—that are found in the National Center for Biomedical Ontology (NCBO) BioPortal. We used a command line version of our tool OntoKeeper [39] to quickly generate scores from the metric suite and then calculated *z* scores to determine how D3X fares in terms of intrinsic quality with other ontologies of the dermatology domain.

## Results

### Development of D3X

**Figure 1:**
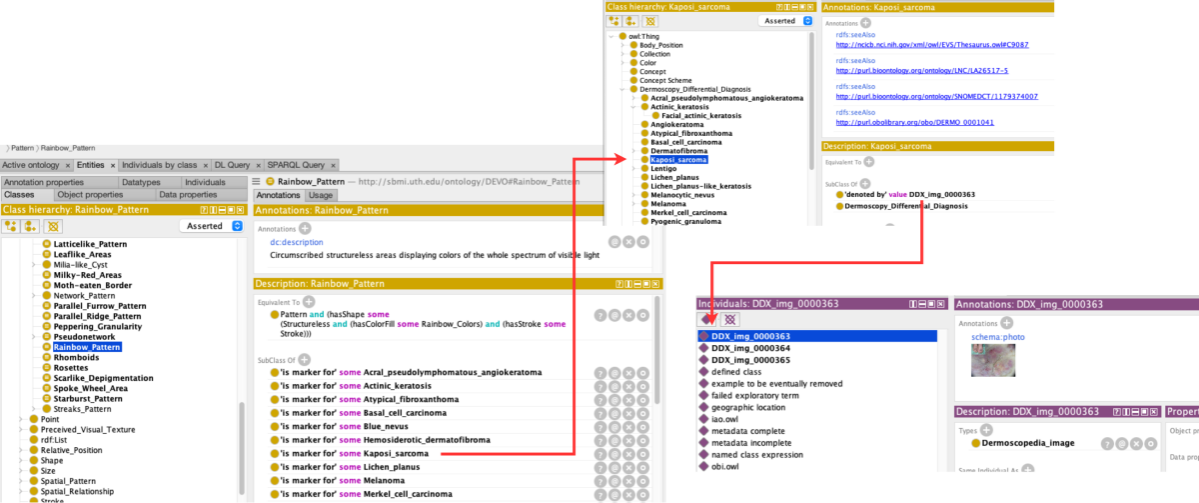
Sample screenshot of the D3X ontology through Protégé showing related metadata and information about Kaposi sarcoma. D3X: dermoscopy differential diagnosis explorer; DDX: differential diagnosis.

**Figure 2:**
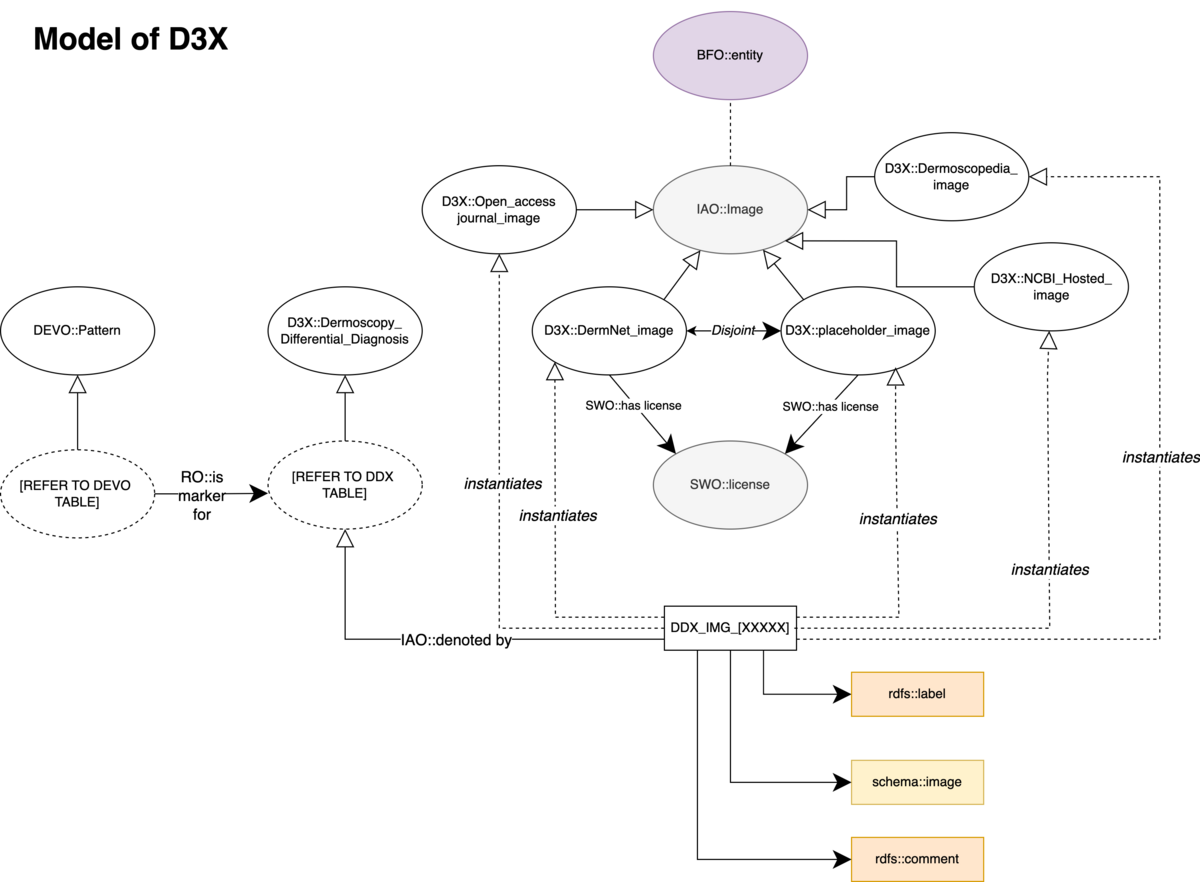
Global overview of the D3X ontology and linked ontologies, including the DEVO, BFO, IAO, and SWO. BFO: basic formal ontology; D3X: dermoscopy differential diagnosis explorer; DEVO: dermoscopy elements of visuals ontology; IAO: information artifact ontology; rdfs: resource description framework schema; RO: relation ontology; SWO: software ontology.

The D3X ontology was encoded in the OWL 2 web ontology language. In terms of the size of the ontology, there are 3041 logical axioms, 1519 classes, 103 object properties, and 20 data properties. Imported image data are encoded as 387 instances. Figure 1 displays a sample series of screenshots showing Kaposi sarcoma, as an example entity, linked to DEVO’s rainbow pattern, standard medical terminologies (eg, Systematized Nomenclature of Medicine–Clinical Terms [SNOMED CT], National Cancer Institute [NCI] Thesaurus), and the open-sourced image example. For ongoing data management, we host the spreadsheet with image data (n=364 images) and the OWL API software code to allow for an automated process of adding new image data. The software will pull the data from the spreadsheet and will add and export a version of our ontology that has the image instance data. Both the spreadsheet and the software are available on our GitHub repository [32]. As more dermoscopy images become available for the public domain, we will include them in our spreadsheet and generate an encoded export with the new instance data. D3X uses our pre-existing work of DEVO and also leverages terminology from the IAO, SWO, and Schema.org. Figure 2 shows a global overview of the D3X ontology and the various linked terminologies that compose the entire model.

Each dermoscopy sample image is represented as a single instance data value with a unique ID (DDX_IMG_[DIGITS]). As an instance data value representing the digital image, it links to the exact file on the web using schema:image from Schema.org. Caption information is used as an annotation for RDF:comment (RDF: resource description framework) and rdf:label (rdf: resource description framework). The instance data value is an instantiation of a specific image class from the following sources: IAO—open access journal images, DermNet images, National Center for Biotechnology Information–hosted images, and Dermoscopedia images. For some of them, the “has licenses” predicate links to a license, signifying that any instance of this image class has some license agreement (eg, Creative Commons). The licensing terminology is derived from the SWO and is hosted on our GitHub repository as an external import.

With D3X, we declared a new dermoscopy differential diagnosis class. This class provides a list of associated diagnoses for skin lesions. Each of the dermoscopy differential diagnosis classes is linked to a pattern from DEVO. The pattern in DEVO ontologically describes each dermoscopic pattern (metaphoric term) using visual elements, such as lines, shapes, colors, and spatial relationships. Table S1 in Multimedia Appendix 1 provides a comprehensive list of the metaphoric patterns listed in DEVO and their corresponding differential diagnoses in D3X. Each pattern in DEVO is linked to its differential diagnoses using OBO’s “is marker for” (eg, angular lines > is a marker for > Lentigo_maligna). Additionally, the instance images described above are linked to the differential diagnoses using “denoted by,” such that each diagnosis is provided with at least one visual example. Lastly, for each of the dermoscopy differential diagnosis classes, there are associated annotations that link the class to the other standardized ontologies like the Medical Dictionary for Regulatory Activities (MedDRA), SNOMED CT, NCI Thesaurus, and LOINC (logical observation identifier names and codes). MedRA covered 63% (n=25) of the classes, while SNOMED CT and NCI Thesaurus covered 53% (n=21) and 55% (n=22) of the classes, respectively. The remaining, like DERMO and LOINC, covered 15% (n=6) and 3% (n=1) of the classes.

### Semiotic Evaluation

Semiotic theory is composed of 3 basic qualities: *syntactic*, *semantic*, and *pragmatic*. Table 1 displays the *z* scores for each of the qualities and subqualities of D3X compared to other publicly available ontologies in the dermatology domain. Examining the *syntactic* quality of D3X (*z*=0.17), while it lacks diverse syntactic *richness* (*z*=–0.74) in comparison with its other domain counterparts, D3X does adhere to syntactic *lawfulness* (*z*=0.49). D3X compares satisfactorily with other ontologies in the *semantic* quality (*z*=0.77). Although the semantic *clarity* subquality was below average than its peers (*z*=–0.91; the ambiguity of labels), D3X does better with semantic *consistency* (*z*=0.56; the number of essentially unique labels) and semantic *interpretability* (*z*=0.65; whether the label has meaning). The *pragmatic* quality is composed of 1 score: *comprehensiveness*, a measure of the coverage of the domain scope of the ontology based on the number of entities encoded, which was nearly below average for D3X (*z*=–0.66). Lastly, the overall score of D3X (*z*=0.58) points to a somewhat better overall quality score than DERMLEX and SPO (*z*=–1.41 and 0.00, respectively). Although DERMO had a slightly higher overall quality than D3X (*z*=0.83), its score is still within 1 SD of the D3X ontology score, so the quality of D3X appears at least comparable to that of the other ontologies within its own domain.

**Table 1:** Semiotic comparison of D3X^a^ to other dermatology ontologies: the DERMLEX^b^, DERMO^c^, and SPO^d^ using *z* scores.

Quality and subquality		Mean (SD)	D3X-z	DERMLEX-z	DERMO-z	SPO-z
**Syntactic**		0.57 (0.11)	0.17^e^	–1.33	0.08	1.09^e^
	Richness	0.26 (0.11)	–0.74	0.62^e^	–0.96	1.08^e^
	Lawfulness	0.87 (0.25)	0.49	–1.50	0.51^e^	0.51 ^e^
**Semantic**		0.85 (0.13)	0.77^e^	–1.38	0.70^e^	–0.08
	Clarity	0.99 (0.01)	–0.91	0.91^e^	0.82^e^	–0.82
	Consistency	0.73 (0.49)	0.56^e^	–1.50	0.43	0.51^e^
	Interpretability	0.87 (0.21)	0.65^e^	0.65^e^	0.16	–1.46
**Pragmatic**		0.11 (0.15)	–0.66	1.44^e^	–0.09^e^	–0.69
	Comprehensiveness	0.11 (0.15)	–0.66	1.44^e^	–0.09^e^	–0.69
Overall score		0.52 (0.03)	0.58 ^e^	–1.41	0.83^e^	0.00

^a^D3X: dermoscopy differential diagnosis explorer.

^b^DERMLEX: dermatology lexicon.

^c^DERMO: human dermatological disease ontology.

^d^SPO: skin physiology ontology.

^e^These values indicate the 2 highest values for each quality and subquality.

## Discussion

### Principal Results and Limitations

D3X is an ontology that connects dermoscopic patterns (metaphoric terms) with differential diagnoses. It is an extension of the DEVO to describe patterns based on their visual elements, which is in turn an extension of the EVO. D3X also leverages terminology from IAO, SWO, Relation Ontology, and Schema.org, and its differential diagnoses are linked to MedDRA, SNOMED CT, and NCI Thesaurus. Using the semiotic theory framework proposed by Burton-Jones et al [38], we measured D3X in comparison with similar publicly available ontologies to assess its intrinsic quality. Our assessment indicates that while comparably better to the other ontologies of the same domain in its overall score, D3X does lack diverse syntactic *richness* and could improve its semantic *clarity* (despite a better overall semantic quality score than its ilk) and pragmatic *comprehensiveness*. Leveraging additional OWL 2 syntactic features could improve the syntactic richness. However, since the purpose of our ontology is to retrieve and aggregate information and metadata about dermoscopic features, some of the more sophisticated OWL 2 features like symmetry, inverse, etc, may not be necessary for our use case. As for the pragmatic score, it might improve over time as we collect more instance data of images to link to our ontology. Further, our assessment was limited to 3 ontologies as there are no other publicly available ontologies that deal solely with a dermatology subject. Additionally, OntoKeeper uses a subset of scores as the social quality (composed of *authority* and *history*), and the pragmatic subscores of *accuracy* and *relevancy* are difficult to compute, so they are not listed in our semiotic analysis [39]. Despite this, the quality scores are sufficient for an application ontology, since the role of this artifact is to aggregate and consolidate skin diagnostic information—an area where it is likely to shine.

The aforementioned evaluation included DERMLEX, DERMO, and the SPO. DERMLEX was originally created by the American Academy of Dermatology to describe dermatological diagnosis and related domain vocabularies, aligned to *International Classification of Diseases, Ninth Revision* (*ICD-9*). However, the upkeep ended in 2009 [22]. DERMO is another ontology that also aims to describe dermatological diseases, but unlike DERMLEX, it is aligned to *International Classification of Diseases, Tenth Revision* (*ICD-10*). The latest version was last released in 2015, according to the NCBO BioPortal record [23]. Not much is known about SPO, other than a presence on NCBO BioPortal and the latest release dating back to 2008 [25]. Compared to these existing works, D3X yields richer semantics and applicability by the OWL2 encoding in EVO and DEVO that describes lesions using primitive visualization elements. Another advantage of this work is the use of semantic web properties of our work, namely the linking of heterogeneous resources (external entities, images, metadata, etc). This allows D3X to be an application-driven artifact that can be integrated into software tools, and other analytical and educational tools. According to researchers, terminologies enriched with semantics will yield opportunities to develop innovative tools and applications [40]. We further discuss our vision in the subsequent sections (see Proposed Web Application and Use Case). Overall, we presume this work provides a richer ontological artifact compared to similar ontologies of the same domain.

**Figure 3:**
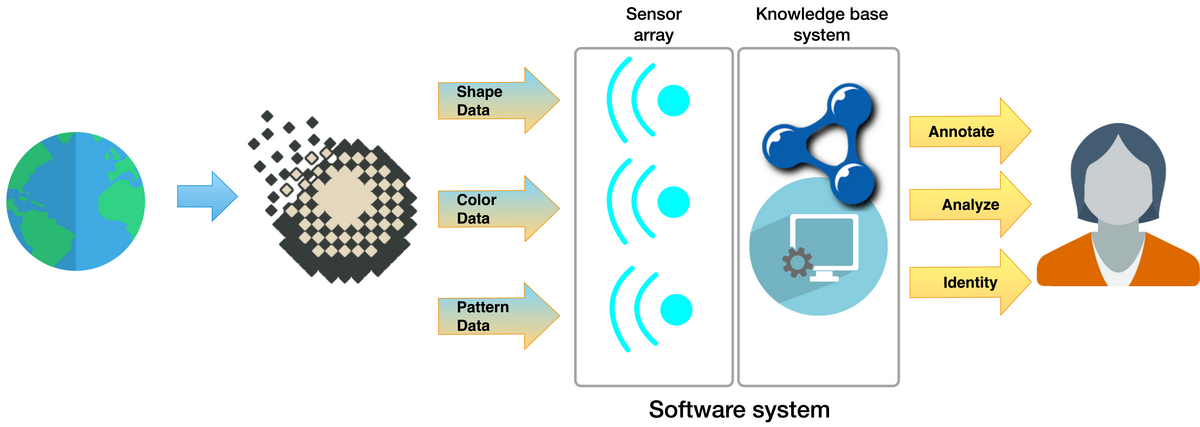
Diagram of a software system using segmented machine learning with a sensor array linked to an ontological knowledge base system. Pixel art icon by DesignContest is licensed under CC BY 4.0. Earth icon by Treetog ArtWork. Globe icon and user female icon by paomedia.

Aside from our aforementioned application use case, this work can advance machine learning models for dermoscopy diagnosis support. There has been some preliminary evidence that machine learning models can be supported or improved by ontologies [41-43]. Potentially, the combined stack of EVO, DEVO, and D3X could augment tools that analyze real-world entities (eg, lesions). In Figure 3, we illustrate a hypothetical example where a software application segments signals from an entity using machine learning in a sensor array to detect shape, color, and pattern data. The structured information from the sensor array could then be linked to an ontological knowledge base system that expresses meaning and context.

### Data Upkeep and Management Plan

**Figure 4:**
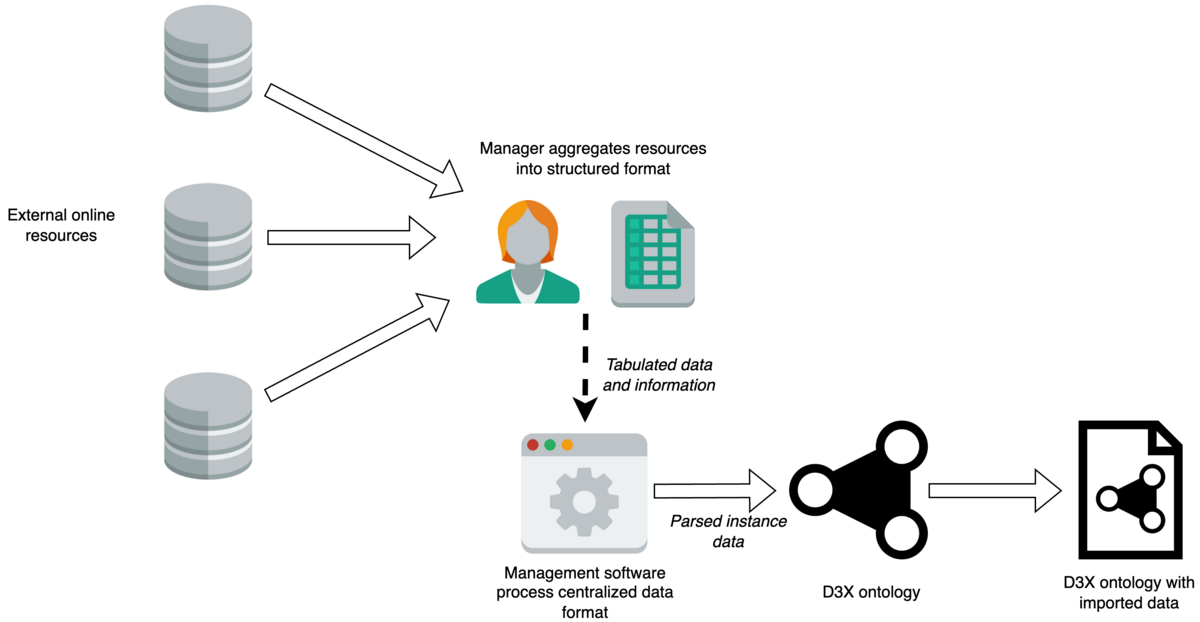
Outline of the D3X ontology data upkeep and management plan. D3X: dermoscopy differential diagnosis explorer. OWL Lite icon and OWL Lite document icon by Picol Team are licensed under CC BY 4.0. File excel icon, database icon, window system icon, user female alt icon by paomedia.

Noted earlier, we produced a basic management system to allow for continued integration of data and information from external sources to be added to D3X. Continued data management is an issue with some ontology and controlled terminology resources. By having this basic management system, we can ensure that D3X will be up to date with little resources and time needed to integrate new diagnostic information and metadata. Figure 4 shows the basic management pipeline, with the tools needed, hosted on our GitHub repository under the ddx_data_management folder. In the aforementioned figure, any new or updated digital resources (images, web page text, knowledge graph, and ontology resources) will be added to a centralized spreadsheet for the human-friendly organization of data for diagnosis information. The management software will import the spreadsheet and parse the data for the D3X ontology. The final output of the software is the D3X ontology with the updated linked information. Future plans could include using shapes constraint language (SHACL) to ensure the quality of the data is validated, and further development of data management software to facilitate ease of use.

### Proposed Web Application and Use Case

**Figure 5:**
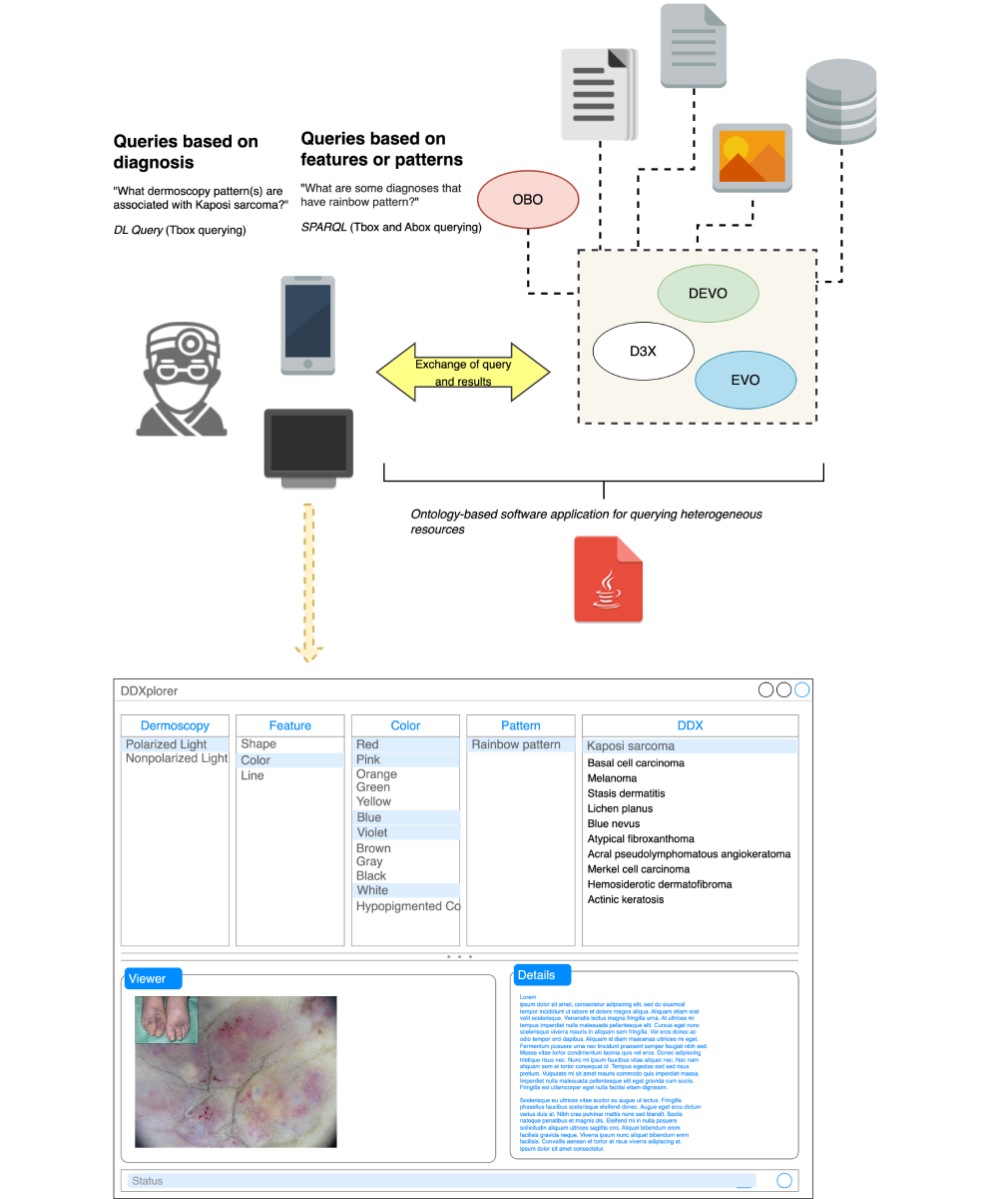
Mock-up of a web application harnessing the D3X ontology to perform queries for differential diagnoses associated with dermoscopic features and patterns, with Kaposi sarcoma as a use case. Doctor Icon by MedicalWP is licensed under CC BY 4.0. Computer icon and text x java icon by Papirus Dev Team are licensed under GNU GPL (version 3.0). Database icon, file text icon, file picture icon, device mobile phone icon by paomedia. Abox: assertion component of a knowledge base; D3X: dermoscopy differential diagnosis explorer; DEVO: dermoscopy elements of visuals ontology; DL: description logic; EVO: elements of visuals ontology; OBO: open biological and biomedical ontology; SPARQL: SPARQL protocol and RDF query language (recursive acronym); Tbox: terminology component of a knowledge base.

For use in clinical practice, an ontology-based software application could be designed using D3X to guide the identification of differential diagnoses. After the user performs dermoscopy on a skin lesion, they can open the web application and select various features about the lesion, which will then suggest differential diagnoses. A mock-up for the use case of Kaposi sarcoma is shown in Figure 5. The user can select options for the first 3 boxes (“dermoscopy,” “feature,” and the chosen feature [eg, “color”], with multi-select functionality available for the latter). The web application would then generate the relevant patterns and a list of differential diagnoses. Thus, if the user indicates that under polarized light, red, pink, blue, violet, and white colors were visualized, this corresponds to a “rainbow pattern,” which is associated with Kaposi sarcoma, among other differential diagnoses. Clicking on each differential diagnosis will then display any relevant dermoscopic images in the “viewer,” as well as a description under “details” with a link to learn more about the condition. By reviewing images and descriptions of these differential diagnoses, this would ideally help the user narrow down the list and identify the most likely diagnosis. This web application was independently reviewed by 2 domain experts who agreed that the format was understandable to the user; they also stated that the information provided would be useful in clinical practice as a quick search for differential diagnoses. Of note, the aforementioned example illustrates a query based on features or patterns, but the web application would also be capable of querying based on diagnoses (eg, “what dermoscopy patterns are associated with Kaposi sarcoma?”). This would be more useful in an educational setting for those who want to gain an understanding of dermoscopic patterns and the features comprising each pattern. Furthermore, we proposed the development of a web application harnessing D3X capable of carrying out the following queries: (1) given dermoscopic features or patterns, output a list of differential diagnoses; and (2) given a differential diagnosis, output associated features and patterns. Along with a description of each differential diagnosis, the application would also display images from DermNet, Dermoscopedia, National Center for Biotechnology Information Hosted, or open-access journals for ease of understanding the relationship between each differential diagnosis and its visual elements. There is a growing body of literature on machine learning models for automated diagnosis of dermoscopic images, such as convolutional neural networks (CNNs) [9]. Both ontologies and CNNs fall under the artificial intelligence umbrella, but ontologies relate to knowledge representation, while CNN is statistical machine learning. These are fundamentally different approaches to power artificial intelligence that are difficult to compare directly. While automated diagnosis via CNNs is a very promising area of study, research has largely focused on the diagnosis of melanoma [44-46], with few studies including pigmented nonmelanocytic lesions [47,48] and largely ignoring nonpigmented lesions. D3X labels dermoscopic patterns of pigmented and nonpigmented lesions, so it may apply to a broader range of patient visits. Additionally, the likelihood of provider acceptance of automated diagnosis systems is unclear. With our proposed web application, providers would be able to input search criteria themselves and see a list of differential diagnoses, rather than a binary output for 1 diagnosis (eg, melanoma) suggested by the machine, which may not be as likely to be accepted by physicians.

In discussion with domain experts, we chose not to integrate diagnostic rules into D3X, as experienced dermoscopy users could assess the list of differential diagnoses fairly quickly and decide on the most likely diagnosis using their clinical expertise. If the user is relatively inexperienced, they may gain more understanding by reading the description of each differential diagnosis, viewing the images, and accessing additional information by clicking “learn more” in the web application. Nevertheless, in the future, it may be useful to integrate diagnostic rules into D3X for more sophisticated suggestions of differential diagnoses (eg, ranking the most to least likely differential diagnoses in a prioritized list). Another limitation is that there are dermoscopic terms and differential diagnoses not included in D3X, given that we built D3X from the terms mentioned in the International Society of Dermoscopy’s third consensus conference [34]. Similarly, while we aimed to include images from a variety of external sources, we acknowledge that they may not be fully representative of all patient skin tones. Our work is only a starting point, as we plan to continue updating D3X and anticipate that its library will become more comprehensive with time.

To conclude, the web application based on D3X has great potential for use in several areas. First, it could be included alongside formal dermoscopy training as a supplementary educational tool for dermatology trainees and providers in other specialties (PCPs, plastic surgeons). Given that providers may require ongoing dermoscopy refresher sessions to feel fully comfortable even after completing an initial training program [17], this web application could be a helpful reference to deepen understanding of dermoscopic patterns associated with different skin conditions. Furthermore, in a clinical setting, providers could quickly query the web application for a list of differential diagnoses after dermoscopic examination of a lesion, which would aid in the identification of their patient’s diagnosis. This may be useful for inexperienced and experienced dermoscopy users alike, as it is intended to augment, not replace, the provider’s clinical reasoning. The next steps include using our proposed interface to build a functional web application. Creating the web application could reveal additional flaws in the design that require clarification, and we would continue to improve aspects of D3X and the web application in an iterative process. After a beta version of the web application is finalized, we would aim to conduct user testing to evaluate the user experience as well as clinical or educational utility among physicians.

### Conclusions

We introduce and discuss the design and development of the D3X ontology as a resource that can link and integrate dermoscopic differential diagnoses and supplementary information with existing ontology-based resources (MedDRA, SNOMED CT, and NCI). We repurposed a previous work of the EVO and DEVO to construct and support D3X, along with other supplementary standardized ontologies, like IAO and SWO. Using the semiotic theoretical framework to compare D3X with other dermatology-related ontologies, its overall quality score was similar to existing ontologies’ scores. One of the outcomes of this work is providing a means to aggregate and link dermoscopic patterns to differential diagnoses, thereby enhancing understanding of dermoscopy for educational and clinical use. This outcome has fueled our next objective in developing a web-based application that can query D3X and fetch the linked information for the user. Currently, D3X and its resources are available on GitHub for public release and use.

## Appendix

Multimedia Appendix 1 Patterns in the dermoscopy elements of visuals ontology and their associated differential diagnoses in the dermoscopy differential diagnosis explorer.

## Abbreviations

CNN: convolutional neural network
D3X: dermoscopy differential diagnosis explorer
DERMLEX: dermatology lexicon
DERMO: human dermatological disease ontology
DEVO: dermoscopy elements of visuals ontology
EVO: elements of visuals ontology
IAO: information artifact ontology
ICD-9: International Classification of Diseases, Ninth Revision
ICD-10: International Classification of Diseases, Tenth Revision
LOINC: logical observation identifier names and codes
MedDRA: Medical Dictionary for Regulatory Activities
NCBO: National Center for Biomedical Ontology
NCI: National Cancer Institute
OBO: Open Biological and Biomedical Ontology
OWL: web ontology language
PCP: primary care physician
rdf: resource description framework
RDF: resource description framework
SHACL: shapes constraint language
SNOMED CT: Systematized Nomenclature of Medicine—Clinical Terms
SPO: skin physiology ontology
SWO: software ontology

## References

[1] Argenziano G, Soyer HP (2001). Dermoscopy of pigmented skin lesions—a valuable tool for early diagnosis of melanoma. Lancet Oncol.

[2] Vázquez-López F, Manjón-Haces JA, Maldonado-Seral C, Raya-Aguado C, Pérez-Oliva N, Marghoob AA (2003). Dermoscopic features of plaque psoriasis and lichen planus: new observations. Dermatology.

[3] Rosendahl C, Tschandl P, Cameron A, Kittler H (2011). Diagnostic accuracy of dermatoscopy for melanocytic and nonmelanocytic pigmented lesions. J Am Acad Dermatol.

[4] Lallas A, Argenziano G, Apalla Z, Gourhant JY, Zaballos P, Di Lernia V, Moscarella E, Longo C, Zalaudek I (2014). Dermoscopic patterns of common facial inflammatory skin diseases. J Eur Acad Dermatol Venereol.

[5] Bafounta ML, Beauchet A, Aegerter P, Saiag P (2001). Is dermoscopy (epiluminescence microscopy) useful for the diagnosis of melanoma? Results of a meta-analysis using techniques adapted to the evaluation of diagnostic tests. Arch Dermatol.

[6] Kittler H, Pehamberger H, Wolff K, Binder M (2002). Diagnostic accuracy of dermoscopy. Lancet Oncol.

[7] Vestergaard ME, Macaskill P, Holt PE, Menzies SW (2008). Dermoscopy compared with naked eye examination for the diagnosis of primary melanoma: a meta-analysis of studies performed in a clinical setting. Br J Dermatol.

[8] Sinz C, Tschandl P, Rosendahl C, Akay BN, Argenziano G, Blum A, Braun RP, Cabo H, Gourhant J, Kreusch J, Lallas A, Lapins J, Marghoob AA, Menzies SW, Paoli J, Rabinovitz HS, Rinner C, Scope A, Soyer HP, Thomas L, Zalaudek I, Kittler H (2017). Accuracy of dermatoscopy for the diagnosis of nonpigmented cancers of the skin. J Am Acad Dermatol.

[9] Weber P, Tschandl P, Sinz C, Kittler H (2018). Dermatoscopy of neoplastic skin lesions: recent advances, updates, and revisions. Curr Treat Options Oncol.

[10] Haliasos EC, Kerner M, Jaimes-Lopez N, Rudnicka L, Zalaudek I, Malvehy J, Hofmann-Wellenhof R, Braun RP, Marghoob AA (2013). Dermoscopy for the pediatric dermatologist part I: dermoscopy of pediatric infectious and inflammatory skin lesions and hair disorders. Pediatr Dermatol.

[11] Westerhoff K, McCarthy WH, Menzies SW (2000). Increase in the sensitivity for melanoma diagnosis by primary care physicians using skin surface microscopy. Br J Dermatol.

[12] Argenziano G, Puig S, Zalaudek I, Sera F, Corona R, Alsina M, Barbato F, Carrera C, Ferrara G, Guilabert A, Massi D, Moreno-Romero JA, Muñoz-Santos C, Petrillo G, Segura S, Soyer HP, Zanchini R, Malvehy J (2006). Dermoscopy improves accuracy of primary care physicians to triage lesions suggestive of skin cancer. J Clin Oncol.

[13] Herschorn A (2012). Dermoscopy for melanoma detection in family practice. Can Fam Physician.

[14] Chappuis P, Duru G, Marchal O, Girier P, Dalle S, Thomas L (2016). Dermoscopy, a useful tool for general practitioners in melanoma screening: a nationwide survey. Br J Dermatol.

[15] Morris JB, Alfonso SV, Hernandez N, Fernández MI (2017). Examining the factors associated with past and present dermoscopy use among family physicians. Dermatol Pract Concept.

[16] Fee JA, McGrady FP, Rosendahl C, Hart ND (2020). Training primary care physicians in dermoscopy for skin cancer detection: a scoping review. J Cancer Educ.

[17] Robinson JK, MacLean M, Reavy R, Turrisi R, Mallett K, Martin GJ (2018). Dermoscopy of concerning pigmented lesions and primary care providers' referrals at intervals after randomized trial of mastery learning. J Gen Intern Med.

[18] Brennan MC, Kabuli MN, Dargan MD, Pinder MR (2022). A short correspondence piece to the editor in chief: the need for increased training in the technique of dermoscopy amongst plastic surgeons and the under recognised value of dermoscopy in the assessment of non-pigmented cutaneous lesions. J Plast Reconstr Aesthet Surg.

[19] Paterlini M (2007). There shall be order. The legacy of Linnaeus in the age of molecular biology. EMBO Rep.

[20] Smith B, Kusnierczyk W, Schober D, Ceusters W (2006). Towards a reference terminology for ontology research and development in the biomedical domain.

[21] Papier A, Chalmers RJG, Byrnes JA, Goldsmith LA, Dermatology Lexicon Project (2004). Framework for improved communication: the Dermatology Lexicon Project. J Am Acad Dermatol.

[22] (2009). Dermatology lexicon. NCBO BioPortal.

[23] Fisher HM, Hoehndorf R, Bazelato BS, Dadras SS, King LE, Gkoutos GV, Sundberg JP, Schofield PN (2016). DermO; an ontology for the description of dermatologic disease. J Biomed Semantics.

[24] Human dermatological disease ontology. NCBO BioPortal.

[25] Skin physiology ontology. NCBO BioPortal.

[26] Benvenuto-Andrade C, Dusza SW, Agero ALC, Scope A, Rajadhyaksha M, Halpern AC, Marghoob AA (2007). Differences between polarized light dermoscopy and immersion contact dermoscopy for the evaluation of skin lesions. Arch Dermatol.

[27] Lin R, Amith M, Zhang X, Wang C, Light J, Strickley J, Tao C (2021). Developing ontologies to standardize descriptions of visual and dermoscopic elements.

[28] Ashton R, Leppard B (2014). Differential Diagnosis in Dermatology. CRC Press.

[29] Noy NF, McGuinness DL (2021). Ontology development 101: A guide to creating your first ontology. Corais.

[30] Egenhofer M, Herring J (1990). A mathematical framework for the definition of topological relations.

[31] Ceusters W (2012). An information artifact ontology perspective on data collections and associated representational artifacts. Studies in Health Technology and Informatics.

[32] Malone J, Brown A, Lister AL, Ison J, Hull D, Parkinson H, Stevens R (2014). The Software Ontology (SWO): a resource for reproducibility in biomedical data analysis, curation and digital preservation. J Biomed Semantics.

[33] Guha RV, Brickley D, Macbeth S (2016). Schema.org: evolution of structured data on the web. Commun ACM.

[34] Kittler H, Marghoob AA, Argenziano G, Carrera C, Curiel-Lewandrowski C, Hofmann-Wellenhof R, Malvehy J, Menzies S, Puig S, Rabinovitz H, Stolz W, Saida T, Soyer HP, Siegel E, Stoecker WV, Scope A, Tanaka M, Thomas L, Tschandl P, Zalaudek I, Halpern A (2016). Standardization of terminology in dermoscopy/dermatoscopy: results of the third consensus conference of the International Society of Dermoscopy. J Am Acad Dermatol.

[35] Yélamos O, Braun RP, Liopyris K, Wolner ZJ, Kerl K, Gerami P, Marghoob AA (2019). Dermoscopy and dermatopathology correlates of cutaneous neoplasms. J Am Acad Dermatol.

[36] Draghici C, Vajaitu C, Solomon I, Voiculescu VM, Popa MI, Lupu M (2019). The dermoscopic rainbow pattern—a review of the literature. Acta Dermatovenerol Croat.

[37] Musen MA, Protégé Team (2015). The Protégé project: a look back and a look forward. AI Matters.

[38] Burton-Jones A, Storey VC, Sugumaran V, Ahluwalia P (2005). A semiotic metrics suite for assessing the quality of ontologies. Data Knowl Eng.

[39] Amith M, Manion F, Liang C, Harris M, Wang D, He Y, Tao C (2019). Architecture and usability of OntoKeeper, an ontology evaluation tool. BMC Med Inform Decis Mak.

[40] Obrst L (2003). Ontologies for semantically interoperable systems.

[41] Mullin S, Zola J, Lee R, Hu J, MacKenzie B, Brickman A, Anaya G, Sinha S, Li A, Elkin PL (2021). Longitudinal K-means approaches to clustering and analyzing EHR opioid use trajectories for clinical subtypes. J Biomed Inform.

[42] Sahoo SS, Kobow K, Zhang J, Buchhalter J, Dayyani M, Upadhyaya DP, Prantzalos K, Bhattacharjee M, Blumcke I, Wiebe S, Lhatoo SD (2022). Ontology-based feature engineering in machine learning workflows for heterogeneous epilepsy patient records. Sci Rep.

[43] Zemmouchi-Ghomari L (2023). Ontology and machine learning: a two-way street to improved knowledge representation and algorithm accuracy.

[44] Rajpara SM, Botello AP, Townend J, Ormerod AD (2009). Systematic review of dermoscopy and digital dermoscopy/ artificial intelligence for the diagnosis of melanoma. Br J Dermatol.

[45] Marchetti MA, Codella NCF, Dusza SW, Gutman DA, Helba B, Kalloo A, Mishra N, Carrera C, Celebi ME, DeFazio JL, Jaimes N, Marghoob AA, Quigley E, Scope A, Yélamos O, Halpern AC, International Skin Imaging Collaboration (2018). Results of the 2016 International Skin Imaging Collaboration international symposium on biomedical imaging challenge: comparison of the accuracy of computer algorithms to dermatologists for the diagnosis of melanoma from dermoscopic images. J Am Acad Dermatol.

[46] Pham TC, Luong CM, Hoang VD, Doucet A (2021). AI outperformed every dermatologist in dermoscopic melanoma diagnosis, using an optimized deep-CNN architecture with custom mini-batch logic and loss function. Sci Rep.

[47] Tschandl P, Kittler H, Argenziano G (2017). A pretrained neural network shows similar diagnostic accuracy to medical students in categorizing dermatoscopic images after comparable training conditions. Br J Dermatol.

[48] Shetty B, Fernandes R, Rodrigues AP, Chengoden R, Bhattacharya S, Lakshmanna K (2022). Skin lesion classification of dermoscopic images using machine learning and convolutional neural network. Sci Rep.

