# Supplementary material for: Dermoscopy Differential Diagnosis Explorer (D3X) Ontology to Aggregate and Link Dermoscopic Patterns to Differential Diagnoses: Development and Usability Study
Source: JMIR Med Inform. 2024 Jun 21;12:e49613. doi: 10.2196/49613 (PMC11226929; doi:10.2196/49613)
Supplement: Multimedia Appendix 1 [file medinform_v12i1e49613_app1.docx]

**Table S1**. Patterns in the Dermoscopy Elements of Visuals Ontology and their associated differential diagnoses in the Dermoscopy Differential Diagnosis Explorer.

| Dermoscopy Elements of Visuals Ontology Patterns | Dermoscopy Differential Diagnosis Explorer Diagnosis |
| --- | --- |
| Angulated_Lines | "Melanoma_on_sun_damaged_skin"; "Lentigo_maligna"; "Lentigo_maligna_melanoma" |
| Annular_Granular_Pattern | "Facial_lentigo_maligna_melanoma"; "Lentigo_maligna" |
| "Asymmetric pigmented follicular openings" | "Lentigo_maligna" |
| Atypical_Pigment_Network | "Dysplastic_nevus"; "Melanocytic_nevus"; "Melanoma" |
| Blotch_Irregular | "Melanoma"; "Seborrheic_keratosis"; "Basal_cell_carcinoma" |
| Blotch_Regular | "Seborrheic_keratosis"; "Basal_cell_carcinoma"; "Dysplastic_nevus" |
| Blue_Gray_Ovid_Nest | "Basal_cell_carcinoma" |
| Blue_Whitish_Veil | "Congenital_melanocytic_nevus"; "Dermatofibroma"; "Reed_nevus"; "Pyogenic_granuloma"; "Basal_cell_carcinoma"; "Seborrheic_keratosis"; "Spitz_nevus"; "Melanoma"; "Angiokeratoma" |
| Branched_Streaks | "Melanocytic_nevus"; "Melanoma" |
| Broadened_Network | "Melanoma" |
| Central_White_Patch | "Dermatofibroma"; "Basal_cell_carcinoma" |
| Cerebriform_Pattern | "Seborrheic_keratosis" |
| Cobblestones | "Congenital_melanocytic_nevus"; "Dermal_nevus" |
| Comedo-like_Openings | "Seborrheic_keratosis" |
| Crypts | "Seborrheic_keratosis" |
| Delicate_Network | "Dermatofibroma" |
| Dots_Irregular | "Basal_cell_carcinoma"; "Squamous_cell_carcinoma"; "Melanoma" |
| Dots_Regular | "Basal_cell_carcinoma"; "Squamous_cell_carcinoma"; "Melanocytic_nevus" |
| Fibrillar_Pattern | "Acral_nevus" |
| Fingerprint_Pattern | "Seborrheic_keratosis"; "Solar_lentigo" |
| Globules_Irregular | "Melanoma"; "Dysplastic_nevus" |
| Globules_Regular | "Melanocytic_nevus"; "Dermatofibroma"; "Basal_cell_carcinoma"; "Clonal_seborrheic_keratosis" |
| Latticelike_Pattern | "Acral_nevus" |
| Leaflike_Areas | "Basal_cell_carcinoma" |
| Milky-Red_Areas | "Dermatofibroma"; "Melanoma"; "Basal_cell_carcinoma"; "Pyogenic_granuloma" |
| Moth-eaten_Border | "Solar_lentigo" |
| Negative_Network | "Melanoma"; "Spitz_nevus"; "Congenital_melanocytic_nevus" |
| Parallel_Furrow_Pattern | "Acral_nevus" |
| Parallel_Ridge_Pattern | "Acral_melanoma" |
| Peppering_Granularity | "Lichen_planus-like_keratosis"; "Melanoma"; "Melanocytic_nevus" |
| Pigment_Network | "Melanocytic_nevus"; "Dermatofibroma"; "Solar_lentigo"; "Melanoma"; "Dysplastic_nevus"; "Ink_spot_lentigo"; "Supernumerary_nipple" |
| Pseudopods | "Reed_nevus"; "Recurrent_nevus"; "Melanoma"; "Spitz_nevus" |
| Pseudonetwork | "Facial_nevus" |
| Radial_Streaming | "Recurrent_nevus"; "Spitz_nevus"; "Melanoma"; "Reed_nevus" |
| Rainbow_Pattern | "Merkel_cell_carcinoma"; "Lichen_planus"; "Kaposi_sarcoma"; "Actinic_keratosis"; "Stasis_dermatitis"; "Acral_pseudolymphomatous_angiokeratoma"; "Basal_cell_carcinoma"; "Hemosiderotic_dermatofibroma"; "Atypical_fibroxanthoma"; "Melanoma"; "Blue_nevus" |
| Rhomboids | "Lentigo_maligna"; "Facial_melanoma" |
| Rim_of_Brown_Globules | "Melanocytic_nevus"; "Spitz_nevus"; "Melanoma"; "Dysplastic_nevus" |
| Rosettes | "Melanoma"; "Squamous_cell_carcinoma"; "Actinic_keratosis" |
| Scarlike_Depigmentation | "Lichen_planus-like_keratosis"; "Melanoma"; "Melanocytic_nevus" |
| Shiny_White_Blotches_and_Strands | "Melanoma"; "Basal_cell_carcinoma"; "Lichen_planus-like_keratosis" |
| Shiny_White_Streaks | "Lichen_planus-like_keratosis"; "Dysplastic_nevus"; "Basal_cell_carcinoma"; "Melanoma"; "Spitz_nevus"; "Dermatofibroma" |
| Spoke_Wheel_Area | "Basal_cell_carcinoma" |
| Starburst_Pattern | "Dysplastic_nevus_with_spitzoid_features"; "Reed_nevus"; "Spitz_nevus" |
| Strawberry_Pattern | "Actinic_keratosis" |
| Typical_Pigment_Network | "Solar_lentigo"; "Dermatofibroma"; "Melanocytic_nevus"; "Ink_spot_lentigo"; "Supernumerary_nipple" |
| "Milia-like Cyst (Cloudy)" | "Seborrheic_keratosis" |
| "Milia-like Cyst (Starry)" | "Seborrheic_keratosis" |
